# The Relationship between Smoking, Raynaud's Phenomenon, Digital Ulcers, and Skin Thickness in the Waikato Systemic Sclerosis Cohort

**DOI:** 10.2478/rir-2022-0014

**Published:** 2022-07-06

**Authors:** Cherumi Silva, Kamal K. Solanki, Douglas H.N. White

**Affiliations:** 1 Rheumatology Department, Waikato Hospital, Private Bag, Pembroke Street, Hamilton, New Zealand.

**Keywords:** digital ulcers, Raynaud's; skin thickness, smoking, systemic, sclerosis

## Abstract

**Objectives:**

Systemic sclerosis (SSc) is a heterogeneous complex autoimmune connective tissue disease with variable presentation as a consequence of multisystem involvement. One of the key features of SSc is Raynaud's phenomenon along with vascular endothelial dysfunction that leads to digital ulcers (DUs). Raynaud's tends to be triggered by decreasing thermal gradient exposure, while stress and smoking also play a role. DUs arising as a consequence of severe Raynaud's and vasculopathy are a major cause of morbidity and disability in SSc. We set out to determine the relationship between smoking, Raynaud's phenomenon, DUs, and skin thickness in our Waikato Systemic Sclerosis cohort.

**Methods:**

The Waikato Systemic Sclerosis (SSc) database was used to extract data. Variables collected included demographics, age of diagnosis, SSc subtypes, age at first non-Raynaud's phenomenon, medications used for treatment of Raynaud's phenomenon or ulcers, and maximal modified Rodnan skin score (mRSS). Raynaud's phenomenon and finger DUs (severity for each over the past week and since diagnosis) and a Scleroderma Health Assessment Questionnaire (SHAQ) visual analog 10 cm scale were collected. The lead rheumatologist completed a physician's assessment of Raynaud's and the disease severity questionnaire.

**Results:**

Of the cohort of 143 patients, 100 patients were eligible to complete the questionnaires. Seventy-five patients returned completed questionnaires. Of these, the majority were female (88%), 52 (69.3%) had limited cutaneous systemic sclerosis (lcSSc), 17 (22.7%) had diffuse cutaneous systemic sclerosis (dcSSc), and 6 (8%) had an overlap syndrome. Thirty-six (48%) had a smoking history (in the time frame of collection of serial data). Mean ± standard deviation (SD) pack-years smoked were 17.11 ± 15.29 years. Thirty-five participants had a history of DUs, with a median of 4 DU (range 1–20). Of 17 patients with dcSSc, 12 (70.6%) had ulcers in comparison with 17 of 52 (32.7%) patients with lcSSc. There was a significant relationship between SSc subtype and the number with ulcers (*X^2^* = 10.1, *P* = 0.007). There was also a significant relationship between physician severity of Raynaud's and presence of ulcers (*t* = 6.1, *P* < 0.001), which was not evident between patients’ severity of Raynaud's and presence of ulcers (*t* = 1.9, *P* = 0.06). On the SHAQ score, smokers had significantly worse Raynaud's phenomenon over the prior week (*t* = 3.08, *P* = 0.03) and were more likely to note DUs over the preceding week, although the latter was not statistically significant (*t* = 1.95, *P* = 0.055). There was no association between smoking and skin thickness as measured by mRSS (*r* = 0.23, *P* = 0.19).

**Conclusion:**

Our study demonstrates that smokers have had worse Raynaud's phenomenon over the past week and they were also more likely to note DUs with a trend toward significance but not statistically significant most likely due to our small sample size. Our study also demonstrated that patients with dcSSc had more ulcers in comparison with lcSSc. This study justifies physicians strongly recommending smoking cessation in patients with SSc.

## Introduction

Systemic sclerosis (SSc) is a heterogeneous complex autoimmune connective tissue disease characterized by enhanced activation of the immune system and vasculopathy, including Raynaud's phenomenon, fibrosis with myofibroblast activation, and endothelial dysfunction.^[1, 2 3]^

In accordance with Le Roy classification there are 2 main types of SSc, namely limited cutaneous systemic sclerosis (lcSSc) and diffuse cutaneous systemic sclerosis (dcSSc).^[4]^ SSc remains a very heterogeneous disease, and it has been noted that this simple scheme does not fit all patients.^[5, 6]^

One of the key manifestations of SSc is Raynaud's phenomenon and digital ulcers (DUs).

Raynaud's is triggered by decreasing thermal gradient exposure, stress, and smoking. DUs as a consequence of severe Raynaud's and vasculopathy are a major cause of morbidity and disability in SSc. Although several potential novel treatments are emerging for Raynaud's as well as DUs, at present there are limited therapeutic options.^[2,7,8]^

Important lifestyle and behavioral changes may assist in preventing disease progression. There are studies showing the impact of smoking on general health issues and the benefits of smoking cessation.^[9, 10]^ Several studies have also looked at the cause–effect relationship between smoking and vascular outcomes in SSc with conflicting results.^[11, 12, 13, 14]^ Harrison et al.^[11]^ demonstrated in a cross-sectional study of 101 patients with SSc in Manchester, United Kingdom, that current smokers were 3–4 times more likely than never smokers to develop digital vascular disease and require treatment (IV Prostanoid, debridement, or amputation). However, interestingly, in this study, past smokers were not at increased risk in comparison with never smokers.

Hudson et al.^[12]^ looked at 606 patients enrolled in the Canadian Scleroderma Research group and concluded that current smoking had a negative, but statistically non-significant, effect on vascular outcomes and variable effects in past smokers. On the other hand, in a cross-sectional study of 98 patients at John Hopkins Medical Centre in Baltimore, Wigley et al.^[13]^ reported that smoking was not associated with a higher risk of DUs. In this study, however, in comparison with others in the literature, there was a higher proportion of non-white patients, which could indicate an association between differing genetic, environmental, and digital vascular insults. Similarly, in a recent study by Jaeger et al.^[14]^ in 2017, smoking was not associated with DUs in the European Scleroderma Trials and Research Group (EUSTAR) cohort of 3023 patients.

We undertook a similar study involving the Waikato SSc cohort, assessing the relationship between smoking and Raynaud's, DUs, and skin thickness modified Rodnan Skin Score (mRSS) in our geographic and differing ethnic setting.

## Methods

### Data Collection

The Waikato Hospital SSc database was used as the primary source for extracting data. This database includes detailed information of patients attended to in a dedicated clinic for patients with SSc as well as overlap syndromes, run by a lead rheumatologist with a special interest in SSc, a specialized nurse, and a registrar. Data have been gathered prospectively since 2005. Patients are seen at either 6-monthly or yearly intervals, and are recruited from surrounding regions as well as locally. The patients seen at each visit are subject to a thorough review of evolutionary history and physical evaluation, as well as laboratory and other relevant investigations, with the information recorded in a standardized form. Data are collected from these patients, with their consent, for the EUSTAR registry as well.

### Ethics Application

New Zealand Health and Disability Ethics committee approval (NZ/1/E2EC09) was obtained for this study. Further locality assessment (RD017039) with review by Te Puna Oranga Maori Consultation Research Review Committee (TPOMRRC) was also conducted prior to undertaking this project. Written or verbal consent was recorded for all patients.

### Variables Collected

These included date of birth, age at scleroderma diagnosis, subtype of scleroderma, age at first non-Raynaud phenomenon, maximal mRSS, and medications used previously for treatment of Raynaud phenomenon or ulcers.

### Questionnaires

Questionnaires (Appendix 1, 2, and 3) were posted (or completed in the clinic) to 100 patients and data were collected between April and August 2017.

Information collected consisted of smoking history (current, past, or never; smoking intensity and duration), Raynaud's phenomenon severity (over the prior week and since diagnosis), DUs (presence or absence as well as total number), and the Scleroderma Health Assessment Questionnaire (SHAQ), a 10 cm visual analog scale looking at 6 variables.^[15, 16]^ A Health Assessment Questionnaire Disability Index (HAQ DI) was not used for this study.^[17]^ The lead rheumatologist was blinded to patients’ questionnaire results and completed a physician's assessment of Raynaud's/disease severity questionnaire (see Appendix 4).

### Statistical Analysis

Microsoft Excel 2010 and IBM SPSS Statistics version 23 (IBM, Armonk, NY, USA) were used to analyze the data. Descriptive statistics were computed as number (n), mean (standard deviation [SD]), or median (range) where appropriate. Associations between categorical variables were assessed with Chi-square or Fisher's exact tests where appropriate. Correlation between continuous variables was assessed with the Pearson correlation coefficient. Independent sample *t*-tests were used to compare means of continuous variables. A *P* value of <0.05 was taken as statistically significant.

## Results

Of the cohort of 143 patients, 23 were deceased, 17 no longer attended the clinic or have migrated overseas, and 3 did not fulfil Le Roy's criteria for SSc or overlap syndrome. Thus, a total of 100 patients were eligible for inclusion in this study.

Of the 100 patients, 25 did not return the questionnaires by the pre-specified date. These patients were thus excluded from the study, leaving 75 patients that met the inclusion criteria.

There was no difference seen between the study participants and the whole cohort, with respect to gender, scleroderma subtype, or clinical manifestations (e.g., lung involvement, cardiac, renal, etc.). However, the study participants were approximately 5 years older, although this was not statistically significant. Thus, participants were considered to be the representative sample of the cohort.

### Demographics and Cohort Characteristics

Of the 75 study participants, 66 (88%) were female. The majority were NZ European (*n* = 57, 76%) and only 3 were Maori (4%). The others were identified as Other European (*n* = 5, 6.7%), Indian (*n* = 3, 4%), Asian (*n* = 3, 4%), Tongan (*n* = 2, 2.7%), and 2 (2.7%) did not specify an ethnicity. The median age was 62 years, with a range of 20–85 years, and the median age of the first non-Raynaud's phenomenon was 54 years (range 26–78 years). The majority had lcSSc (*n* = 52, 69.3%), 17 (22.7%) had dcSSc, and 6 (8%) had an overlap syndrome. The mean maximum mRSS was 8.09 (SD 7.48).

### Smoking, Digital Ulcers and Max-mRSS

Thirty-six (48%) had a smoking history in the timeframe of study data collection. Mean ± SD pack-years smoked was 17.11 ± 15.29 years, with a median of 11 years and a range of 0.1–50.0 pack-years. The median age at starting smoking was 16.5 years (range 7–25 years) while the median age at stopping smoking was 39.5 years (range 20–68 years).

Thirty-five patients reported ulcers, although 3 could not recall how many. There were a total of 163 ulcers thus recorded in 32 patients. Of 17 patients with dcSSc, 12 patients (70.6%) had ulcers in comparison with only 17 of 52 (32.7%) patients with lcSSc. Out of 6 (50%) patients with overlap syndrome, 3 reported ulcers. There was a significant relationship between SSc subtype and the number with ulcers (*X^2^* = 10.1, *P* = 0.007).

Of 35 patients with DUs, 21 were smokers (which included current plus ever smoked while having SSc) (60%) and 14 were non-smokers (40%). Four patients were current smokers at the time of data lock on August 31 (despite earlier advice to quit smoking), and 17 were past smokers. There was a near significant increased DUs in smokers, but this did not reach statistical significance (*X^2^* = 3.77, *P* = 0.052).

In our study, there was no relationship between quantity of cigarettes smoked and maximal mRSS (*r* = −0.23, *P* = 0.19).

### Raynaud's Phenomenon and Ulcer Severity

Table 1 below shows Raynaud's phenomenon scores by the physician and patients. There was a significant association between the physician's and patients’ scoring of Raynaud's severity over the past week (*r* = 0.25, *P* = 0.028), as well as since diagnosis (*r* = 0.52, *P* < 0.001).

**Table 1:** Physician and patients scores of Raynaud's phenomenon (smokers and non-smokers)

**Raynaud's severity (0–10)**	**Mean**	**SD**	**Smokers Mean (SD)**	**Non-smokers Mean (SD)**
Physician scoring of RP severity over the prior week	3.51	2.38	3.97 (2.52)	3.08 (2.18)
Physician scoring of RP severity since diagnosis	5.92	2.49	6.47 (2.61)	5.41 (2.29)
Patient scoring of RP severity over the prior week	4.42	2.99	5.11 (3.29)	3.79 (2.58)
Patient scoring of RP severity since diagnosis	5.55	2.82	5.72 (3.37)	5.38 (3.26)

SD, standard deviation; RP, Raynauds phenomenon; ACE, Angiotensin converting enzyme.

The mean physician score of DU severity over the past week was 1.07 (2.29) while it was 3.07 (3.52) since diagnosis. The mean patient score of ulcer severity over the past week was 2.71 (3.49). Although there was a significant relationship between physician severity of Raynaud's and presence of DUs (*t* = 6.1, *P* < 0.001), this was not evident between patients’ severity of Raynaud's and presence of DUs (*t* = 1.9, *P* = 0.06).

### Drugs used for Raynaud's and/or Digital Ulcers

Data on medication use for Raynaud's and/or ulcers were available for all 75 study participants (see Table 2). Of these, 17 (22.7%) were on no medication and 24 (32%) were on calcium channel blockers (CCBs) (dihydropyridine type) alone. Several patients were on combination therapy as indicated below, including 1 patient who was on a CCB, IV Prostanoid, Phosphodiesterase type 5 inhibitor (PDE5), and Endothelin Receptor Antagonist (ERA, Macitentan); the latter as part of a drug trial at one stage.

**Table 2:** Medications used for treating Raynaud's and/or DUs

**Drugs**	***n* (%)**
No medication	17 (22.7)
CCB	24 (32)
SSRI	3 (4)
CCB and SSRI	8 (10.7)
CCB and PDE5	5 (6.7)
CCB and IV Prostanoid	5 (6.7)
SSRI and PDE5	1 (1.3)
SSRI and IV Prostanoid	1 (1.3)
CCB, IV Prostanoid, and SSRI	4 (5.3)
CCB, SSRI, and PDE5	1 (1.3)
CCB, IV Prostanoid, and PDE5	2 (2.7)
CCB, SSRI, and ACE inhibitor	1 (1.3)
CCB, SSRI, and Topical Nitrate	1 (1.3)
CCB, IV Prostanoid, PDE5, and ERA	1 (1.3)
CCB, SSRI, PDE5, and IV Prostanoid	1 (1.3)

CCB, calcium channel blocker; ERA, endothelin receptor antagonist; PDE5, phosphodiesterase type 5 inhibitor; SSRI, selective serotonin re uptake inhibitor; DU, digital ulcers.

### SHAQ Scores (10 cm Visual Analog Scale), Raynaud's, and Pack-years Smoked

All participants completed SHAQ scores, graded on a 10 cm visual analog scale, as depicted in Table 3.

**Table 3:** Scleroderma Health Assessment Questionnaire (SHAQ) in past week

**SHAQ variable past week**	***n***	**Minimum**	**Maximum**	**Mean**	**SD**	**Smokers Mean (SD)**	**Non-smokers Mean (SD)**
Severity of pain	75	0	88	31.53	27.84	35.00 (29.53)	28.33 (26.15)
Severity of intestinal symptoms	75	0	91	18.09	25.94	22.72 (29.44)	13.82 (21.74)
Severity of shortness of breath	75	0	100	21.83	27.53	28.22 (30.14)	15.92 (23.77)
Severity of Raynaud's	75	0	91	28.45	27.88	38.22 (31.23)	19.44 (21.01)
Severity of finger ulcers	75	0	91	11.75	25.01	17.50 (29.72)	6.44 (18.55)
Severity of disease	75	0	100	33.03	29.47	33.81 (29.71)	32.31 (29.62)

SHAQ, scleroderma health assessment questionnaire; SD, standard deviation.

Smokers had significantly worse SHAQ score for Raynaud's phenomenon over the prior week (*t* = 3.08, *P* = 0.03), but the physician scoring of Raynaud's phenomenon over the past week (*t* = 1.65, *P* = 0.104) or since diagnosis (*t* = 1.88, *P* = 0.065) was not influenced by smoking.

However, there was a significant relationship seen between pack-years smoked and SHAQ-RP severity (*t* = 3.62; df = 50.86; *P* = 0.0006). Likewise, there was also a significant relationship between pack-years smoked and the number of ulcers (Mann–Whitney *U* 254.5; *P* = 0.04).

Further, smokers were more likely on the SHAQ questionnaire to complain of shortness of breath over the prior week (*t* = 1.97, *P* = 0.053) as well as note DUs over the preceding week, although the latter did not reach statistical significance (*t* = 1.95, *P* = 0.055).

Other SHAQ variables, namely, the pain score and gastrointestinal symptoms score, were not affected by smoking (*t* = 1.04, *P* = 0.303; *t* = 1.49, *P* = 0.14, respectively).

## Discussion

This is the first study to date in New Zealand looking at the relationship between smoking and clinical features of SSc, namely Raynaud's phenomenon, DUs, and skin thickening. The characteristics of the population studied are s imilar to cohorts of SSc elsewhere, consisting of predominantly a female population with a median age of onset in the mid-50s.^[12, 18, 19]^ Our cohort had 48% past smokers but only 5.3% current smokers (i.e., at date of data evaluation), unlike the study by Hudson et al.,^[12]^ where, in a cohort of 606 patients, there was a similar number of past smokers (42%) but 16% current smokers.

In our study, there was a higher frequency of DUs (statistically significant) in patients with dcSSc as opposed to other subtypes, in keeping with other studies.^[20]^ Smokers had increased DUs, but this did not reach statistical significance, which probably reflects our relatively small sample size.

Caramaschi et al.^[21]^ have demonstrated that several factors including smoking history were significantly associated with ischemic DUs. Interestingly, smoking was shown be negatively associated with skin thickening, which is similar to the association with other conditions such as Ulcerative Colitis and Sarcoidosis.^[22, 23]^ This was also demonstrated by Gyger et al.^[24]^ in 2012 in the Canadian Scleroderma cohort of 606 patients, where smoking was significantly associated with less severe skin scores. They postulated that this effect was possibly due to the known effect of smoking in promoting premature skin atrophy by reducing collagen production.

There was a positive correlation between physician and patient scoring of Raynaud's severity since diagnosis, which was statistically significant. This was not noted for ulcers over the past week. This is most likely due to the physician's recall of patient's past histories and lack of awareness of recent events.

Our study did demonstrate a statistically significant association between physician scoring of Raynaud's and presence of DUs that were not present in the patients’ questionnaires, possibly reflecting individual patients’ perception of disease severity.

In our study, the majority of patients were on dihydropyri-dine type of CCBs for treatment of Raynaud's and/or DUs, in keeping with other studies where this is often the first-line treatment.^[25, 26]^ A meta-analysis by Thompson et al.[27] has previously demonstrated a reduction in severity and frequency of episodes of Raynaud's phenomenon in patients with SSc. There was a variety of other medications used in our patients for treatment of Raynaud's phenomenon or DUs, often combination therapy, in keeping with other studies.^[26, 28, 29]^

We acknowledge several limitations with our study. First, the sample size (given the low population of our country) was small, which could have reduced our ability to detect small effect sizes. Other limitations include recall bias for both the physician and the patients completing the questionnaires. We also did not include nail fold capillaroscopy data, which have been shown to be predictive of vasculopathy and organ damage in SSc.^[30]^ However, there are some strengths that should be mentioned. First, only the lead rheumatologist scored disease severity and was blinded to the results of the patient questionnaires. Second, the study was undertaken over winter, thereby attempting to capture most episodes of Raynaud's and/or DUs. Also, all SSc patients have had access to our online help system and hence they were seen and assessed promptly to reduce recall bias, as well as for optimal documentation.

## Conclusion

Our study has demonstrated results in keeping with international studies.^[11, 12]^ Smokers have worse Raynaud's phenomenon and increased DUs, as well as increased shortness of breath.

As there is a high prevalence of patients in our cohort with a smoking history and as there is evidence to suggest a negative influence of smoking on various health aspects of SSc, the results of this study provide useful information for the patients who visit our clinic.

Measures should be put into place in the follow-up clinics of SSc to counsel patients and to encourage them to quit smoking, in order to decrease the incidence and severity of Raynaud's and DUs, which have been shown in other studies to be associated with significant morbidity.
