# Supplementary material for: The Relationship between Smoking, Raynaud's Phenomenon, Digital Ulcers, and Skin Thickness in the Waikato Systemic Sclerosis Cohort
Source: Rheumatol Immunol Res. 2022 Jul 6;3(2):84–9. doi: 10.2478/rir-2022-0014 (PMC9524819; doi:10.2478/rir-2022-0014)
Supplement: Supplementary file 1 [file rir-03-084_sm.pdf]

Supplementary Materials

Appendix 1

Smoking questionnaire

Have you ever smoked?

Yes ☐ No ☐

If yes, then at what age did you
- Start?
- Stop?
- Approximately how many cigarettes a day did you smoke? (If the response is "roll your own," please write approximately how many grams of tobacco a week you smoked).

Appendix 2

Patients assessment of severity of Raynaud's disease

In the past week, how severe was your Raynaud's disease on a scale of 0 to 10, where 10 is very severe and 0 is not severe?
Overall, since you were diagnosed with scleroderma, how severe was your Raynaud's disease on a scale of 0 to 10, where 10 is very severe and 0 is not severe?
Have you ever had ulcers on your fingers related to your Raynaud phenomenon?

Yes ☐ No ☐

If yes, how many ulcers have you had?
If yes, in the past week, how severe were your finger ulcers on a scale of 0 to 10, where 10 is very severe and 0 is not severe?

Appendix 3

Scleroderma Health Assessment Questionnaire (SHAQ)

We are also interested in learning whether or not you are affected by pain because of your illness.
How much pain have you had because of your illness **IN THE PAST WEEK?**

PLACE A MARK ON THE LINE TO INDICATE THE SEVERITY OF THE PAIN.

NO PAIN — VERY SEVERE PAIN

0 —————————————— 100

IN THE PAST WEEK, how much have your intestinal problems interfered with your daily activities?

PLACE A MARK ON THE LINE TO INDICATE THE LIMITATION OF ACTIVITY.

INTESTINAL PROBLEMS DO NOT LIMIT ACTIVITIES — VERY SEVERE LIMITATION

0 —————————————— 100

IN THE PAST WEEK, how much have your breathing problems interfered withyour daily activities?

PLACE A MARK ON THE LINE TO INDICATE THE LIMITATION OF ACTIVITY.

BREATHING PROBLEMS DO NOT LIMIT ACTIVITIES — VERY SEVERE LIMITATION

0 —————————————— 100

IN THE PAST WEEK, how much has Raynaud's interfered with your daily activities?

PLACE A MARK ON THE LINE TO INDICATE THE LIMITATION OF ACTIVITY.

RAYNAUD'S DOES NOT LIMIT ACTIVITIES — VERY SEVERE LIMITATION

0 —————————————— 100

IN THE PAST WEEK, how much have your finger ulcers interfered with your daily activities?

PLACE A MARK ON THE LINE TO INDICATE THE LIMITATION OF ACTIVITY.

FINGER ULCERS DO NOT LIMIT ACTIVITIES — VERY SEVERE LIMITATION

0 —————————————— 100

Overall, considering the amount of pain, discomfort, and limitations in your daily life and other changes in your body and life, how severe would you rate your disease today?

PLACE A MARK ON THE LINE TO INDICATE THE LIMITATION OF ACTIVITY.

NO DISEASE VERY SEVERE LIMITATION

0 100

Appendix 4

Physician's assessment of severity of Raynaud's disease

In the past week, how severe would you rate the patient's Raynaud's disease on a scale of 0–10, where 10 is very severe and 0 is not severe?

Overall, since diagnosed, how severe is this patient's Raynaud's disease on a scale of 0–10, where 10 is very severe and 0 is not severe?

In the past week, how severe would you rate the patients finger ulcers on a scale of 0–10, where 10 is very severe and 0 is not severe?

Overall, since diagnosed, how severe would you rate the patients finger ulcers on a scale of 0–10, where 10 is very severe and 0 is not severe?

Drugs used over course of illness to treat Raynaud's disease

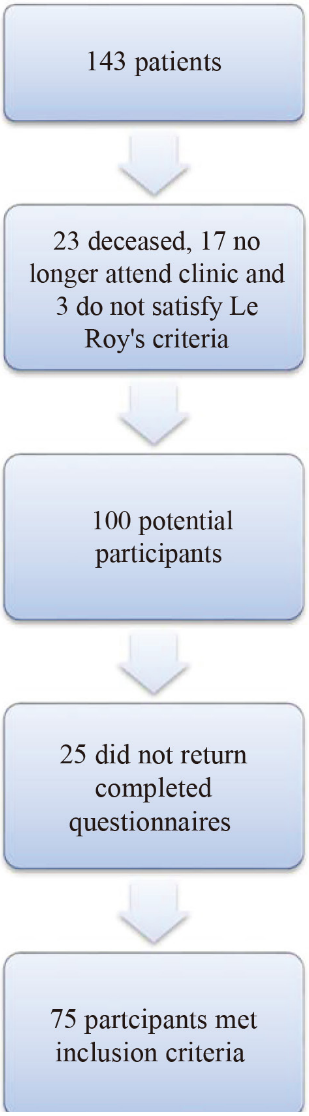

Figure A1: Data Flow of assessment with Questionnaires
